# Major Bleeding in Adults Undergoing Peripheral Extracorporeal Membrane Oxygenation (ECMO): Prognosis and Predictors

**DOI:** 10.1155/2022/5348835

**Published:** 2022-01-15

**Authors:** Tung Phi Nguyen, Xuan Thi Phan, Tuan Huu Nguyen, Dai Quang Huynh, Linh Thanh Tran, Huy Minh Pham, Tu Ngoc Nguyen, Hieu Trung Kieu, Thao Thi Ngoc Pham

**Affiliations:** ^1^Department of Intensive Care, Ho Chi Minh City University of Medicine and Pharmacy, Ho Chi Minh City, Vietnam; ^2^Intensive Care Unit, Vinmec International Hospital, Ho Chi Minh City, Vietnam; ^3^Intensive Care Unit, Cho Ray Hospital, Ho Chi Minh City, Vietnam; ^4^Department of Hematology, Cho Ray Hospital, Ho Chi Minh City, Vietnam

## Abstract

**Background:**

Major bleeding has been a common and serious complication with poor outcomes in ECMO patients. With a novel, less-invasive cannulation approach and closer coagulation monitoring regime, the incidence of major bleeding is currently not determined yet. Our study aims to examine the incidence of major bleeding, its determinants, and association with mortality in peripheral-ECMO patients.

**Method:**

We conducted a single-center retrospective study on adult patients undergoing peripheral-ECMO between January 2019 and January 2020 at a tertiary referral hospital. Determinants of major bleeding were defined by logistic regression analysis. Risk factors of in-hospital mortality were determined by Cox proportional hazard regression analysis.

**Results:**

Major bleeding was reported in 33/105 patients (31.4%) and was associated with higher in-hospital mortality [adjusted hazard ratio (aHR) 3.56, 95% confidence interval (CI) 1.63–7.80, *p* < 0.001). There were no significant difference in age, sex, ECMO indications, ECMO modality, pre-ECMO APACHE-II and SOFA scores between two groups with and without major bleeding. Only APTT >72 seconds [adjusted odds ratio (aOR) 7.10, 95% CI 2.60–19.50, *p* < 0.001], fibrinogen <2 g/L [aOR = 7.10, 95% CI 2.60–19.50, *p* < 0.001], and ACT >220 seconds [aOR = 3.9, 95% CI 1.20–11.80, *p*=0.017] on days with major bleeding were independent predictors.

**Conclusions:**

In summary, major bleeding still had a fairly high incidence and poor outcome in peripheral-ECMO patients. APTT > 72 seconds, fibrinogen < 2 g/L were the strongest predicting factors for major bleeding events.

## 1. Background

Extracorporeal membrane oxygenation (ECMO) has been widely accepted as a treatment for life-threatening cardiac and pulmonary failure [1, 2]. Nevertheless, patients undergoing ECMO are predisposed to bleeding through a variety of mechanisms including underlying critical conditions prompting ECMO initiation, comorbidities, multi-organ dysfunction, and ECMO system itself [3–5]. Major bleeding was a common and serious complication during ECMO, with an overall prevalence of 55–60% and a potential association with a worse outcome in two retrospective cohorts on a total of 281 patients from 2010 to 2013 [6, 7]. Major bleeding on day 1 was independently associated with a two-to-three-fold increase in 90-day and in-hospital mortality risk in ECMO patients [8]. Besides, patients with bleeding complications had a longer ECMO duration and a lower rate of successful ECMO weaning [8].

Risks of bleeding complications in ECMO patients have been linked to various factors including anticoagulation use and APACHE III score [6]. Another significant independent predictor was high activated partial prothrombin time (APTT) 24 hours prior to bleeding, with a three-fold higher risk of bleeding for APTT ≥ 70 seconds [6] and an 11% increase in bleeding risk for each increment of 10 seconds [9]. Morever, ECMO procedure itself is also accompanied by an elevated bleeding risk, with a three-time increase in venoarterial ECMO [9] and post-surgical ECMO [6], and two-time in central VA ECMO [10]. Nonetheless, these studies, to a certain extent, still showed some discrepancies in bleeding determinants, as bleeding risks were associated with VA ECMO type or central cannulation technique in some studies [7, 9, 11] but not in others [6, 12].

Relentless efforts have been made with a view to minimizing bleeding complications in ECMO patients. In terms of technical improvements, biocompatible coatings for cannulas, tubing set and oxygenator membrane have been developed in order to limit activation of coagulation factors, which decreased requirement for blood transfusion, and possibly, rate of major bleeding events [13]. Other developments of ECMO systems are smaller system size and optimization of tube size for better blood flow rate. In addition, ELSO has published a recent 2014 highly-consented guideline for anticoagulation and blood product transfusion [14], which should facilitate a closer monitoring and a better care for ECMO patients in clinical daily practice.

Given those aforementioned current advances in cannulation technique and ECMO systems to mitigate hemorrhage complications, incidence of major bleeding in ECMO patients should be lower by now, with an incidence of 26.5% in a cohort of 34 ECMO cases from 2015 to 2017 [12]. A retrospective study on 164 Dutch patients between 2010 and 2017 reported an overall rate of major bleeding of 45% [9], which were lower than two previous studies [6, 7]. In all these studies, however, a markedly high proportion of ECMO patients received central cannulation (25–39%) and post-surgical or post-cardiotomy ECMO (26–36%) [6, 7, 9], which should overestimate bleeding rate in ECMO patients. The incidence of major bleeding in nonsurgical peripheral ECMO patients, hence, remains questionable.

Since 2019, all ECMO patients in our ICU were cannulated peripherally by skilled intensivists using percutaneous Seldinger technique, and were tested routinely for anti-Xa, aPTT and ACT, as recommended by our institutional protocol and ELSO guidelines, to closely monitor and promptly adjust unfractioned heparin dose. We conducted this study to (1) investigate incidence of major bleeding; (2) identify determinants of major bleeding; and (3) examine the association between major bleeding and survival to hospital discharge in peripheral ECMO patients.

## 2. Patients and Methods

### 2.1. Study Design and Patients

We conducted a single-center retrospective cohort study on adults (age ≥18 years) who received venovenous (VV), venoarterial (VA), or venoarterial-venous (VAV) peripheral ECMO between January 2019 and January 2020 in a medical-surgical 28-bed Intensive Care Unit (ICU) with annual admission of over 100 ECMO cases at Cho Ray Hospital, a tertiary referral hospital in South Vietnam. Exclusion criteria were (1) patients receiving ECMO for less than 24 hours, (2) those with pulmonary embolism treated with fibrinolytic therapy right before or during ECMO, and (3) those with inadequate hemostasis test monitoring, namely ACT, APTT, and anti-Xa. This study was approved by the Research Ethics Committee of Ho Chi Minh City University of Medicine and Pharmacy (IRB-VN01002), and by the Research Ethics Committee of Cho Ray Hospital (No.122/HĐĐĐ).

### 2.2. Cannulation Technique and ECMO Configuration

In our center, acutely ill patients were managed promptly and ECMO was indicated at the discretion of intensivists. In all ECMO cases performed in our ICU, cannulas were placed bedside by experienced intensivists using percutaneous Seldinger technique under ultrasound guidance to minimize duration of cannula insertion and risk of bleeding. Prior to cannula insertion, we measured vascular diameters using ultrasound to select appropriate case-by-case cannula size that could optimize blood flow yet reduce vascular damage and bleeding. ECMO systems in our ICU included a Rotaflow console, PLS membrane, and cannulas from Maquet, Getinge group, Sweden. Cannula size ranged from 15 to 17 Fr for arterial cannulas and from 21 to 25 Fr for venous cannulas.

### 2.3. Bleeding Complication

We defined bleeding complications as bleeding noted by a physician or nurse in medical records. Major bleeding is clinically overt bleeding associated with any of the two following criteria: (1) a fall in hemoglobin (Hb) concentration ≥20 g/L, blood loss ≥20 ml/kg, or need of ≥10 ml/kg packed red blood cells (PRBC) transfusion over 24 hours; and (2) retroperitoneal, pulmonary, or central nervous system bleeding, or bleeding that requires surgical intervention [14].

Major bleeding was managed mainly by lowering unfractioned heparin dose and transfusing blood products at the discretion of intensivists, and then further specific treatment was based on bleeding sites. Bleeding at cannulation sites or surgical incision was managed by sewing then applying a pressure bandage while internal bleeding required more invasive interventions. Thoracostomy with chest tube placement, for example, was used to treat hemothorax. In case of upper gastrointestinal bleeding, endoscopy was performed to locate bleeding sites and apply hemoclips if indicated, followed by proton-pump inhibitor infusion. Intra-abdominal hemorrhage was controlled by surgery and vascular embolization if necessary.

### 2.4. Transfusion and Anticoagulation Practice

In daily clinical practice, we followed our institutional guidelines for indications and targets of blood products transfusion developed by our hospital. PRBC transfusion was indicated to maintain a Hb concentration >80 g/L, or >100 g/L in case of persistent hypoxemia (SaO_2_ < 88%) and/or high lactate levels despite optimal ECMO and ventilator settings and vasopressor use. Platelet transfusion was used to keep platelet count >80 × 10^9^/L in patients without bleeding or >100 × 10^9^/L in those with active bleeding. Fresh frozen plasma was indicated in the presence of at least one of three following scenarios: (1) anti-thrombin III level <50%, (2) a decrease in anti-thrombin III level induced by heparin resistance, or (3) INR >1.5. Cryoprecipitate transfusion was transfused when fibrinogen concentration was <2 g/L.

Unfractioned heparin (UFH) was given to all ECMO patients, with its dose titrated regularly based on hemostasis parameters to maintain ACT within 180–220 seconds, APTT within 45–80 seconds, and anti-Xa within 0.3–0.7 UI/ml according to our local protocol and ELSO Anticoagulation Guideline [14]. Heparin efficacy was monitored by ACT, APTT, and anti-Xa. While ACT and APTT were tested routinely at a 6-hour interval, anti-Xa level, a costly test that was not covered by insurance and unavailable in weekends, was monitored only once daily during weekdays.

### 2.5. Data Collection

We extracted data including patients' demographics, medical history, anticoagulant use, and diagnoses from medical records by a structured data collection form. Pre-ECMO arterial blood gas values, lactate concentrations, SOFA, and APACHE-II scores were collected along with bleeding complications, infections, acute kidney injury, and patient outcomes (successful ECMO weaning, mortality in ECMO, survival to hospital discharge, and in-hospital mortality). Daily hemostatic parameters were also collected, including lowest platelet count, highest INR, lowest fibrinogen, highest APTT, and ACT values. Indications for ECMO (pulmonary, circulatory, or ECPR), ECMO modalities (VV, VA, VAV), time of ECMO initiation, time of cannula removal, and patient outcomes were also collected.

Acute kidney injury (AKI) was determined after admission and during ECMO by KDIGO 2012 criteria. [15] Nosocomial infections were defined by definitions of Centers for Disease Control and Prevention/National Nosocomial Infections Surveillance system. [16] ECMO-related nosocomial infections were defined as nosocomial infections which occurred later than 24 hours after ECMO initiation and prior to 48 hours after ECMO discontinuation. [17, 18].

### 2.6. Statistical Analysis

Notwithstanding the heterogeneity of ECMO indications, and consequently, risks of bleeding in VV- and VA-ECMO patients, we did not perform our analysis separately on each subgroup for small subgroup sample sizes, which would underpower statistical tests and result in a lack of observations in multivariate analysis. Although VA ECMO patients are supposedly more susceptible to bleeding than VV ECMO patients [7, 9], two previous retrospective studies still showed comparable major bleeding rates between these two subgroups [6, 12].

Hemostasis parameters were classified into two groups of in-target and off-target by recommended targets for ECMO patients, namely 220 seconds for ACT, 100 × 10^9^/L for platelet count, 1.2 for INR, and 2 g/L for fibrinogen [14]. Given the lack of a recommended target for APTT, an optimal cut-off of APTT to predict major bleeding in our patients was defined by Youden's index, which is calculated as (sensitivity + specificity) −1, with a higher result yielding a better diagnostic accuracy [19].

We first employed univariate logistic regression analysis to examine the associations between possible determinants and major bleeding, and subsequently selected significant determinants with *p* value <0.05 into multivariate analysis to identify independent predictors. Daily-monitored hemostasis tests, namely platelet count, INR, fibrinogen, ACT, and APTT, were included into logistic regression as binary variables: on-target and off-target. On the other hand, anti-Xa, an inadequately tested coagulation parameter, was excluded from logistic regression analysis. Selecting significant determinants from logistic analysis into prognostic models, we determined the best model predicting major bleeding by Bayesian Model Averaging (BMA) method [20].

Categorical variables were reported in frequency and proportion. Continuous variables were reported in mean and standard deviation, or median and interquartile range (IQR). Differences between two groups with and without major bleeding were tested by either Chi-squared or Fisher's exact tests for categorical variables, and student *t*-test or Mann–Whitney *U* test for continuous variables when appropriate. Survival-to-discharge rates of those two groups were compared by log-rank test, illustrated by a Kaplan-Meier plot. Predictors of survival to hospital discharge were defined in a Cox proportional hazard regression model, first univariate then multivariate, including major bleeding as a potential predictor. The proportional hazards (PH) assumption was checked by scaled Schoenfeld residuals.

## 3. Results

From January 2019 to January 2020, 117 adult patients underwent ECMO at the research center. Twelve patients were excluded from the study for ECMO for less than 24 hours (2 patients), pre-ECMO or peri-ECMO fibrinolytic therapy to treat acute pulmonary embolism (2 patients), and inadequacy of hemostasis test results (8 patients). Among 105 patients entering the final analysis, 61 (58.1%) had VA-ECMO, 38 (36.2%) had VV-ECMO, and 6 (5.7%) had VAV-ECMO. Demographics and clinical characteristics of our patients are shown in Tables 1 and 2.

### 3.1. Thrombosis Events

In our 105 ECMO patients, we did not record any patients with thrombosis during ECMO, including cerebral embolism, pulmonary embolism, myocardial infarction, or other site embolisms. We have 3 cases (3%) that need to change the ECMO oxygenator before 7 days and 7 cases (7%) that need to change the ECMO oxygenator before 14 days due to increasing transmembrane pressure or decreasing oxygenator function. In 81 successful ECMO weaning patients, we documented 15/81 cases (18.5%) of venous and arterial thrombosis following ECMO cannulation and only 2 patients with limb ischemia required surgery to remove thrombus afterward.

### 3.2. Bleeding Events

**Figure 1:**
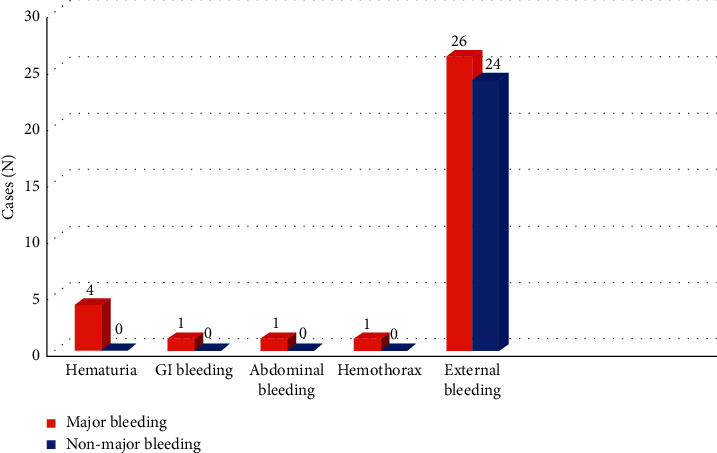
Bleeding sources.

Clinically overt bleeding was found in 57/105 patients (54.3%), among whom 33 (57.9%) had major bleeding. All of seven internal bleeding cases were major bleeding, including hematuria (4/7, 57.1%), gastrointestinal bleeding (1/7, 14.3%), abdominal bleeding (1/7, 14.3%), and hemothorax (1/7, 14.3%). Major bleeding was defined in 26/50 (52%) of external bleeding cases – bleeding at cannula site, surgical incision, nasal or oral cavities (Figure 1).

There were no significant differences in age, sex, ECMO indications, ECMO modality, APACHE-II, and SOFA scores between two groups with and without major bleeding (Table 1). Compared to those without major bleeding, patients with major bleeding required more blood products transfusion (all *P* values <0.001).

Among 347 days with coagulation test results obtained twice a day, major bleeding was observed in 27 days (7.8%). Days with major bleeding had a lower platelet count (88 (71–153) vs 129 (99–185) × 10^9^/L), lower fibrinogen (2.33 (1.14–2.89) vs 4.75 (3.35–6.14) g/L), but higher INR (2.01 (1.38–3.09) vs 1.18 (1.07–1.40)), higher ACT (218 (198–235) vs 193 (182–206) seconds) and higher APTT (77.8 (60.0–96.0) vs 49.2 (42.1–60.0) seconds) than days without major bleeding (all *P* values < 0.001). Among days with major bleeding, hemostasis targets were unattainable in 16/27 days (62.3%) for platelet count, 12/27 days (44.5%) for fibrinogen, 24/27 days (88.9%) for INR, 10/27 days (37.0%) for ACT, and 17/27 days (62.9%) for APTT with our optimal cut-off.

### 3.3. Major Bleeding and Changes in Hemostatic Tests

A cut-off of APTT of 72 seconds best discriminated between patients with and without major bleeding, with a sensitivity of 0.67, a specificity of 0.906, and Youden index of 0.573. APTT >72 seconds, hence, was defined as off-target values and associated with a higher risk of major bleeding (univariate analysis: OR = 16.4; 95% CI: 7.1–40.4, *P* < 0.001). In univariate analysis, other off-target hemostasis values also increased risk of major bleeding with an OR (95% CI) of 5.0 (2.2–11.8) for platelet <100 × 10^9^/L, 9.8 (3.3–41.8) for INR >1.2, 17.5 (6.9–44.9) for fibrinogen <2 g/L, and 5.48 (2.2–12.9) for ACT >220 seconds (all *P* values <0.001). Only fibrinogen <2 g/L, ACT >220 seconds, and APTT >72 seconds on days with major bleeding, however, were independently associated with an increase in major bleeding risk in multivariate analysis (Table 3).

Of five significant determinants from univariate analysis, only APTT >72 seconds, fibrinogen <2 g/L, and ACT >220 seconds were selected by BMA method to produce two best models of similar accuracy to predict major bleeding in ECMO patients, with APTT and fibrinogen included in Model 1 (AUROC = 0.85) and all three predictors included in Model 2 (AUROC = 0.87).

### 3.4. Major Bleeding and Mortality

**Figure 2:**
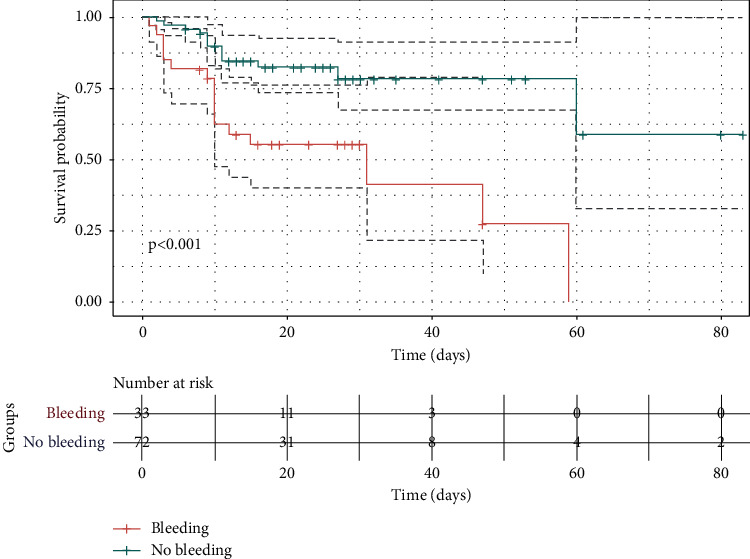
Kaplan–Meier survival estimates of patients with and without major bleeding.

30/105 patients (28.6%) died during their hospital stay, among whom 24 patients (80.0%) died during ECMO. Patients who died were older, more severe, and more prone to acute kidney injury and major bleeding (Table 2). Compared to those without major bleeding, patients with major bleeding had significantly higher ECMO and in-hospital mortality (39.4% vs 15.3%, *P* < 0.013; and 51.5% vs 18%, *P* < 0.001 respectively).  Figure 2 further illustrates cumulative rate of survival to hospital discharge of those two groups with and without major bleeding.

**Figure 3:**
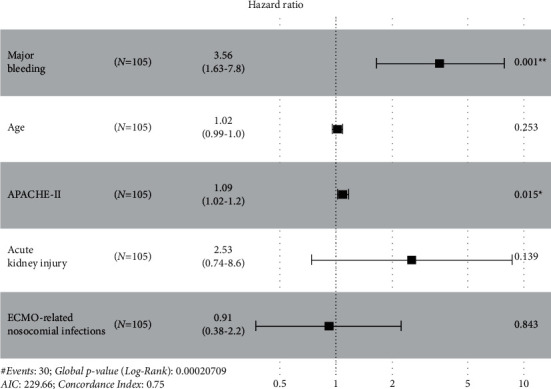
Factors independently associated with in-hospital mortality.

In univariate analysis, mortality risk increased in older patients (OR, 95% CI for each increment in age: 1.05, 1.02–1.09, *P*=0.003), patients with a higher severity score (OR, 95% CI for each increment in APACHE II and SOFA scores: 1.13, 1.06–1.23, *P*=0.001; and 1.23, 1.06–1.44, *P*=0.007, respectively), major bleeding (OR, 95% CI: 4.80, 1.97–12.25, *P* < 0.001), ECMO-related nosocomial infections (OR, 95% CI: 2.60, 1.10–6.30, *P*=0.03), and AKI (OR, 95% CI: 4.50, 1.40–20.10, *P*=0.02). Nevertheless, only major bleeding and APACHE-II remained as independent predictors of in-hospital mortality in multivariate survival analysis using Cox's regression model (aHR, 95% CI: 3.56, 1.63–7.80, *P*=0.001; and 1.09, 1.02–1.20, *P*=0.015, respectively) (Figure 3).

## 4. Discussion

Our study found that major bleeding was still a common and probably fatal complication among ECMO patients, with an incidence of 31.4% (33/105 patients) and an association with a nearly four-fold higher risk of in-hospital death. It should be noted that risks of major bleeding was not associated with ECMO modalities or disease severity but only off-target coagulation tests, with three independent determinants yielding two similarly accurate predictive models, namely APTT > 72 seconds, ACT > 220 seconds, and fibrinogen <2 g/L.

The incidence of major bleeding in our study was lower than 45% from a recent Dutch study [9], and much lower than overall rates of 60% and 56% in two previous retrospective studies [6, 7]. Compared to previous studies by Aubron and Mazzefi, our ECMO patients also had fewer days with major bleeding (7.8% vs 15.1% and 10.0%, respectively) [6, 7], which could be attributable to a significant proportion of patients at greater risk of bleeding–those receiving central or post-cardiotomy/post-surgical ECMO. Yet, after exclusion of those high-risk subgroups, our incidence of major bleeding among peripheral-ECMO patients was still, to some extent, lower than those previous studies (45% [7] and 37% [9]). This signifies a trend in the reduction of major bleeding rates in ECMO patients, thanks to innovations in cannulation technique and ECMO system, closer monitoring of anticoagulation effects with rapid but accurate tests, and a stricter regime of anticoagulation dose adjustment.

The predominance of external bleeding in this study (26/33 patients with major bleeding, 78.8%) was consistent with previous findings by Aubron [6] and Oude Lansink-Hartgring [9]. On the other hand, the primary source of bleeding in a study by Mazzefi was chest and gastrointestinal hemorrhage, which might explain a higher total and individual amount of blood products transfusion than in our study [7]. Interestingly, while all those previous studies reported a small number of cases with, a fatal complication, intracranial hemorrhage [6, 7, 9], none was found in our study. Besides, our study found a lower incidence of intracranial and gastrointestinal bleeding than ELSO's report [2], which could be a result of our institutional regime of early blood products transfusion and concomitantly close anticoagulation monitoring.

There was no statistically significant difference in pre-ECMO factors corresponding to major bleeding complications in this study. Patients with myocardial infarction on dual antiplatelet therapy did not have higher rates of major bleeding; however, this can be attributed to the fact that there were few patients with myocardial infarction in this study, with most on carefully regulated anticoagulant dosing and early hemostatic regulation.

In our study, the incidence of major bleeding might not differ between VA and VV ECMO patients, which further lends support to previous findings by Aubron [6] and Arnouk [12]. Compared to VV ECMO, VA ECMO patients are theoretically supposed to have a greater risk of bleeding for its complexity of cannulation technique, more severe multiorgan failure, and coagulation disorder. Our finding is contradictory to two studies by Mazzefi [7] and Oude Lansink-Hartgring [9], the latter of which demonstrated that VA ECMO was an independent predictor of major bleeding with a HR of 2.89 [9]. In that study, however, the majority of VA ECMO patients (64/101, 63.3%) were cannulated centrally [17], which involved sternotomy and direct incision of right atrium and aorta [22], and therefore could be a confounding factor in the association between VA ECMO and major bleeding if not adjusted [10]. Our comparable rates of major bleeding between VA and VV ECMO suggest that current peripheral cannulation by easy-to-perform percutaneous Seldinger technique might lessen bleeding risks in VA ECMO patients compared to the early dawn of ECMO era.

On the majority of days with major bleeding, coagulation tests, most frequently INR, platelet count, and APTT, failed to attain target ranges. The strong association between the above-target APTT and major bleeding in our study is consistent with several previous studies [6, 9, 12], which highlights the significance of APTT in UFH dose monitoring and titration in order to prevent major bleeding in ECMO patients. Moreover, our optimal APTT cut-off to predict major bleeding was in agreement with cut-offs in other studies [6, 12], and lies within therapeutic range recommended in ELSO guideline [14]. Another independent determinant of major bleeding in this study, fibrinogen, which has not been fully examined in some previous studies [6, 9, 12], could be a potential predictor in clinical practice. Despite its normal transient decrease in the first few hours of ECMO [23], low fibrinogen concentration is present in severe coagulation disorders commonly found in ECMO patients, such as acute liver failure or disseminated intravascular coagulation, which yields the need of routine monitoring of fibrinogen level and further investigation in its association with major bleeding.

Our finding of no relationship between platelet count and major bleeding is comparable to previous results by Aubron and Oude Lansink-Hartgring [6, 9]. In a study by Arnouk on 34 ECMO patients, platelets <150 × 10^9^/L at baseline significantly increased risk of major bleeding by 5.6 times; that association, however, was not adjusted in multivariate analysis [12]. One plausible explanation could be our regimen of early blood products transfusion to maintain platelet count above 80 × 10^9^/L. Moreover, platelets should not drop significantly during ECMO since median platelet count was still above 80 × 10^9^/L on both days with and without bleeding even with a less intensive blood transfusion regime than our study [6, 9].

ACT and APTT are two hemostatic tests widely used in clinical practice to monitor UFH dose, and therefore, their correlation with major bleeding should reflect the effect of anticoagulation use during ECMO. It should be noted that, however, changes in APTT and ACT could also be present in other conditions, and they correlated poorly with UFH dosage or anti-Xa, a more specific assay [24–28]. In contrast to the ACT and aPTT, the anti-Xa assay is specific to the anticoagulant effect of UFH and is not influenced by coagulopathy, thrombocytopenia, or dilution [14]. However, an anti-Xa value is also affected by technical errors from the photo-optical method, such as hyperbilirubinemia, hemolysis, and antithrombin deficiency [29]. In our previous research, we showed that the APTT value is strongly correlated with anti-Xa value, particularly in patients with normal antithrombin levels [30]. ACT, on the other hand, was poorly correlated with UFH dose, whether there is AT deficiency or not [30]. Still, the superiority of anti-Xa to APTT and ACT in correlation with UFH dose has not reached a concrete conclusion with some discordant results [31, 32], which suggests that a combination of multiple tests should be more reliable in predicting UFH dose than a single test.

Major bleeding was an independent risk factor of in-hospital mortality in our study, which is in line with other previous studies [6, 9]. In another study by Mazzefi, major bleeding was associated with increased in-hospital mortality in univariate but not in multivariate analysis [7]. However, major bleeding might have an effect on long-term survival, with a marginally higher survival rate at 90 days in patients without major bleeding (64.9% vs 46.7%, *P*=0.08) [7]. An indirect measure of major bleeding, the amount of blood products transfused, was linked to a higher mortality in some studies [7, 33, 34]. These results underline the importance of early prevention, recognition, and management of major bleeding in an attempt to improve outcomes in ECMO patients.

Our study had several limitations. First, providing that our sample size was not large enough, we did not perform subgroup analysis in VA ECMO and VV ECMO patients, who might have different bleeding risk profiles. Second, our study lacks information on daily assessment of multiorgan dysfunction, particularly liver function, which might contribute to risks of both hemorrhage and mortality. Third, our single-center study was conducted at a tertiary referral hospital in South Vietnam, which might, to a certain extent, produce selection bias. Finally, our study also had information bias, a nature of retrospective studies, as a small subset of patients were excluded for a lack of coagulation tests.

## 5. Conclusion

In conclusion, major bleeding was a common and serious complication with poor outcome in ECMO patients. Off-target APTT, ACT and fibrinogen were three independent predictors of major bleeding, suggesting routine monitoring and adjustment of UFH dose and transfusion of blood products to maintain those tests in therapeutic ranges.

## Figures and Tables

**Table 1:** Clinical characteristics of ECMO patients with and without major bleeding.

Characteristics	All patients (*n* = 105)	Patients with major bleeding (*n* = 33)	Patients without major bleeding (*n* = 72)	*P*
Age (yrs)	39 [30; 52]	39 [30; 54]	40 [31; 51]	0.923
Gender, male	51 (48.5)	13 (39.4)	38 (52.8)	0.287
BMI				
*Comorbidities*				
Hypertension	9 (8.5)	3 (9.0)	6 (8.3)	0.12
Diabetes mellitus	2 (1.9)	0 (0)	2 (0)	
Coronary artery disease	2 (1.9)	1 (0.95)	1 (0.95)	
Coagulation disorder	0 (0)	0 (0)	0 (0)	
Malignancy	0 (0)	0 (0)	0 (0)	
Gastroduodenal ulcer	1 (0.95)	0 (0)	1 (0.95)	
*Diagnosis*				
Acute myocarditis	42 (40.0)	14 (42.4)	28 (38.9)	0.352
ARDS	40 (38.1)	9 (27.2)	31 (43.1)	
AMI	12 (11.4)	5 (15.2)	7 (9.7)	
Others	11 (10.5)	5 (15.2)	6 (8.3)	
*ECMO indications*				
Cardiac	60 (57.1)	22 (66.7)	38 (55.5)	0.081
Pulmonary	44 (41.9)	10 (30.3)	34 (47.2)	
ECPR	1 (1.0)	1 (3.0)	0 (0.0)	
*ECMO modalities*				
VA	61 (58.1)	23 (69.7)	38 (52.8)	0.238
VV	38 (36.2)	8 (24.2)	30 (41.7)	
VAV	6 (5.7)	2 (6.1)	4 (5.5)	
Pre-ECMO APACHE-II score	20 [15; 25]	22 [19; 26]	19 [15; 23]	0.163
Pre-ECMO SOFA score	11 [9; 13]	11 [10; 14]	11 [9; 13]	0.550
Pre-ECMO cardiac arrest	13 (12.3)	6 (18.2)	7 (9.7)	0.337
*Coagulation profile*				
Platelet count (× 10^9^/L)	190 [117; 258]	142 [68; 246]	198 [139; 258]	0.037
INR	1.4 [1.1; 1.6]	1.4 [1.2; 2.4]	1.3 [1.1; 1.5]	0.09
APTT (s)	39 [30; 55]	42 [36; 61]	36 [29; 54]	0.04
Fibrinogen (g/L)	3.5 [3.4; 4.1]	3.5 [2.7; 4.9]	3.7 [2.5; 4.5]	0.82
*Blood products transfusion during ECMO*				
RBC (ml)	126 [58–230]	230 [131–245]	101 [44–126]	<0.001
PLT (ml)	31 [0–137]	167 [45–321]	0 [0–44]	<0.001
FFP + cry (units)	2 [1; 8]	8 [4; 14]	0 [0; 4]	<0.001
*ECMO complications*				
Nosocomial infections	42 (40.0)	17/33 (51.5)	25 (34.7)	0.159
AKI	77 (73.3)	23/33 (69.4)	59 (81.8)	0.270
*Outcomes*				
Successful ECMO weaning	81 (77)	20 (60)	61 (85)	0.013
ECMO mortality	24 (22.8)	13 (39.4)	11 (15.3)	0.013
ICU mortality	29 (27.6)	16 (48.4)	13 (18.0)	0.002
In-hospital mortality	30 (28.6)	17 (51.5)	13 (18)	<0.001

Data are presented as *n* (%) categorical variables and median (interquartile range) for nonparametric variables. ARDS, acute respiratory distress syndrome; AMI, acute myocardial infarction; RBC, red blood cell; PLT, platelet count; FFP, fresh frozen plasma; Cry, cryoprecipitate; AKI, acute kidney injury was defined by KDIGO criteria; ECMO, extracorporeal membrane oxygenation.

**Table 2:** Clinical characteristics of survivors and nonsurvivors.

Characteristics	Survivors (*n* = 75)	Nonsurvivors (*n* = 30)	*P*
Age (yrs)	38 [29; 49]	51 [35; 57]	0.002
Male gender	33 (44.0)	18 (60.0)	
BMI	22.2 [20.7; 24.6]	21.8 [20.4; 23.4]	0.313
*Comorbidities*			
Hypertension	6 (8.0)	3 (10.0)	
Diabetes mellitus	2 (2.7)	0 (0.0)	
Coronary artery disease	1 (1.3)	1 (3.3)	
Coagulation disorder	0 (0.0)	0 (0.0)	
Malignancy	0 (0.0)	0 (0.0)	
Gastroduodenal ulcer	1 (1.3)	0 (0.0)	
*Diagnosis*			
Acute myocarditis	30 (40.0)	12 (40.0)	
ARDS	33 (44.0)	7 (23.3)	
AMI	5 (6.7)	7 (23.3)	
Others	7 (9.3)	4 (13.3)	
*ECMO indications*			
Cardiac	40 (53.3)	22 (73.3)	
Pulmonary	34 (45.3)	8 (26.7)	
ECPR	1 (1.3)	0	
*ECMO modalities*			
VA	39 (52.0)	22 (73.3)	
VV	30 (40.0)	8 (26.7)	
VAV	6 (8.0)	0	
Pre-ECMO APACHE-II score	19 [14; 23]	24.5 [20; 27]	<0.001
Pre-ECMO SOFA score	11 [8; 13]	13 [10; 15]	0.004
Pre-ECMO cardiac arrest	13/75 (17.3)	0	
*Complications*			
Nosocomial infections	46 (61.3)	21 (70.0)	
AKI	50 (66.7)	27 (90.0)	
Thrombosis events			
Major bleeding	16 (21.3)	17 (56.7)	
Number of days with ECMO			
Length of hospital stay (days)			

Data are presented as *n* (%) for categorical variables and median (interquartile range) for nonparametric variables. ECPR, Extracorporeal cardiopulmonary resuscitation; VA, venoarterial; VV, venovenous; VAV, venoarteriovenous; APACHE-II, Acute Physiologic Assessment and Chronic Health Evaluation-II; SOFA, Sequential Organ Failure Assessment.

**Table 3:** Predictors of major bleeding in logistic regression analysis.

Predictors	Univariate analysis			Multivariate analysis		
Platelet count <100 × 10^9^/L	5.00	2.20–11.80	<0.001	1.84	0.64–5.25	0.25
INR >1.2	9.79	3.30–41.80	<0.001	3.30	0.91–15.81	0.09
Fibrinogen <2 g/l	17.50	6.90–44.90	<0.001	6.10	1.90–20.40	0.002
ACT >220s	5.48	2.20–12.90	<0.001	3.90	1.20–11.80	0.017
APTT >72s	16.40	7.10–40.40	<0.001	7.10	2.60–19.50	<0.001

aOR, adjusted odds ratio; CI, confidence interval; APTT, activated partial thromboplastin time; ACT, activated clotting time.

## Data Availability

Data are available on request to the authors. A summary of relevant information will be published with the manuscript.
